# Risk Information Seeking Behavior in Disaster Resettlement: A Case Study of Ankang City, China

**DOI:** 10.3390/ijerph17197352

**Published:** 2020-10-08

**Authors:** Jia Shi, Xiangnan Hu, Xuesong Guo, Cuihong Lian

**Affiliations:** School of Public Policy and Administration, Xi’an Jiaotong University, Xi’an 710049, China; jiashi@xjtu.edu.cn (J.S.); huxiangnan@stu.xjtu.edu.cn (X.H.); cuihonglian1988@163.com (C.L.)

**Keywords:** information seeking behavior, risk communication, disaster resettlement, structural equation model

## Abstract

In 2011, the Chinese government launched a disaster mitigation and preparedness program called the Resettlement of South Shaanxi (RSS). Due to the wide geographical scope and complex interests, the possibility of conflicts was increased during and after resettlement. Efficient risk communication improves the supply of information about risks and meets the risk-related information needs of individuals. Using the risk information seeking and processing (RISP) model, this research applied a structural equation model and survey with a structured questionnaire to study ways to improve risk communication in disaster resettlement. A total of 616 valid questionnaires were provided by study respondents in resettlement sites in Ziyang County, Ankang City, Shaanxi Province. The results indicated the following: (1) the public’s information seeking behavior relies more on village committees and village officials than other channels. Emerging information channels, such as Weibo and WeChat (social media applications in China), do not play leading roles in disseminating risk information. (2) There are differences between the information channels used by residents and the channels that residents believe the most. (3) Relevant channel beliefs, information sufficiency, perceived hazard characteristics, and self-efficacy directly influence risk information seeking behavior. However, the capacity to gather information has non-significant direct influences on information seeking behavior. (4) Perceived hazard characteristics and self-efficacy drive risk information seeking behavior in both direct and indirect ways through information sufficiency.

## 1. Introduction

Disaster resettlement is a recovery and preparedness measure for people that cannot live in their original location due to prevailing risks [1]. Due to the complex interests in these resettlements, significant economic, physical, and social impacts can heighten the possibility of social risks and conflicts during and after resettlement. For example, in some parts of China, residents may perceive different probability levels associated with disaster-related risks and their potential consequences, and may have different levels of self-confidence about how to protect themselves from loss. Residents mainly resort to shangfang (petitioning high-level government authorities to appeal perceived unfair actions), jingzuo (sit-ins on the doorsteps of government buildings or on highways and railways to obstruct transportation), demonstrations, parades, or propagandizing their appeals by refusing to be resettled [2,3,4]. These problems continue to challenge a comprehensive understanding of disaster resettlement.

Communication and interactions inside and outside of communities are critical components of disaster management [5,6,7,8]. Risk communication is a critical way to reduce risk, and the efficiency of risk communication depends on improving the influx of information and understanding individual needs. However, only a few studies have explored what motivates individuals to participate in risk communication interactions and to seek information about the risks associated with them. To fill this research gap, a new direction has emerged in risk communication research [9,10,11,12], focusing on risk information seeking behavior at the individual level. Several studies have focused on individuals seeking risk information. The most commonly used model is the risk information seeking and processing (RISP) model because it provides predictors related to individual information and risk behavior status [11].

This study contributes to disaster resettlement research. Disaster resettlement has been discussed in the literature [1,13,14,15,16]; however, few studies have centered on China. China often suffers from floods [17]. More than 1000 flood disasters have occurred in China, in locations such as the Nen River, Songhua River, and the Yangtze River. This number has exceeded the world average, causing serious losses of life and property [18]. More than 100 large and medium cities are inundated by floods, causing approximately USD 17 billion in economic losses each year [19]. In 2011, the Chinese government launched a disaster mitigation and preparedness program called the Resettlement of South Shaanxi (RSS), which is the largest disaster resettlement in the country’s history. This program was launched to assist the cities of Ankang, Hanzhong, and Shangluo, located in the hinterland of the Qinling Mountains, south of Shaanxi Province, China. This area has experienced serious flood- and earthquake-related disasters. In addition to the serious impact of geological disasters, the RSS involves a wide range of areas and complex interests and relationships. Insufficient risk communication on specific issues, such as livelihoods and resettlement plans, can become a fuse that triggers resentment during resettlement. This may also increase the possibility of social risks and conflicts. Therefore, based on the RISP model and structural equation model (SEM), this study examined individual risk information seeking, and explored two research goals to help improve risk communication in the disaster resettlement process. The first goal was to describe risk information seeking behaviors in the RSS. The second was to identify the main determinants of RSS risk information seeking behavior. 

This paper has six sections. Following this introduction, Section 2 reviews information seeking models and key model components, and then proposes the research hypotheses and a conceptual model of risk information seeking behavior. Section 3 introduces the data and method. Section 4 discusses the research findings. The last section presents our conclusion and discusses the limitations, future studies, and implications of this research.

## 2. Theoretical Lens and Hypotheses

### 2.1. Information Seeking Behavior in Disasters

**Figure 1:**
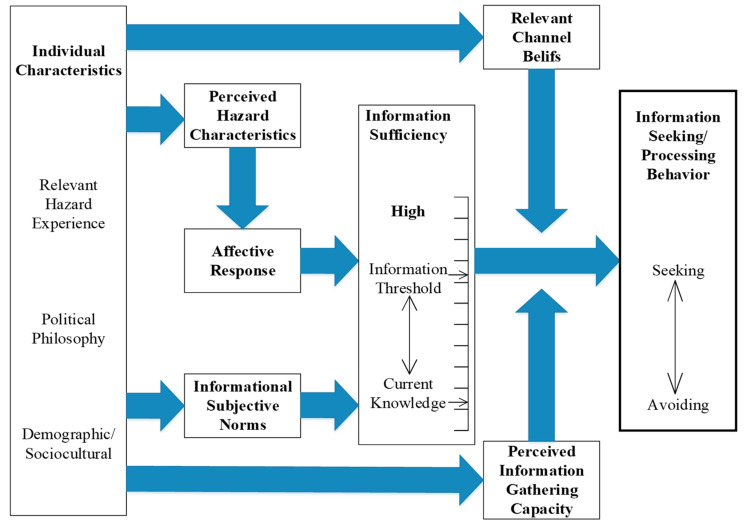
Model of risk information seeking and processing from the original model by Griffin et al. (1999) [11].

The risk information seeking and processing (RISP) model was proposed by Griffin et al. The model focuses on individual information search and processing behavior in a risk environment [11,20]. The RISP model disentangles the social, psychological, and communicative factors that drive risk information seeking, and examines the impact of risk perceptions on how individuals seek and manage risk information (see Figure 1). It also offers a framework to depict the key factors that predispose individuals to seek and process relevant risk information in a more systematic manner. In this framework, risk information seeking and processing are intentions which refer to individuals’ active seeking or avoiding of information. Responses are rated from the weakest to the strongest [20] and measured using continuous scales [11]. Concepts in the model were derived from the heuristic–systematic model (HSM) [21] and the theory of planned behavior (TPB) [22,23]. Based on this, risk perception research [24] and media theories [25] also further enrich the model.

Figure 1 shows the RISP model, in which Griffin et al. proposed three direct determinants of information seeking behavior: information insufficiency, perceived information-gathering capacity, and relevant channel beliefs (public’s beliefs about different information channels) [11]. The model also considers indirect predictors, including individual characteristics, perceived hazard characteristics, informational subjective norms, and affective responses. In 2008, Griffin et al. adjusted the affective responses and informational subjective norms to be direct predictors of information seeking behavior [10]. Other scholars have enriched the original RISP model with new predictors. Kahlor et al. simplified the model to four variables [26]. Ter Huurne added social–psychological variables to the original RISP model, developed a new framework called the framework of risk information seeking (FRIS) [27], and proposed informational subjective norms and affective responses as two new direct motivators of information seeking behavior [28]. In more recent studies, scholars integrated risk perception into this framework. In 2012, Kellens et al. explored the main determinants of information seeking behavior. The results showed that risk perception, efficacy beliefs, and perceived hazard knowledge were positively correlated with information seeking behavior. They also focused on the mediating role of information needs [29]. Liao et al. showed that channel beliefs, current knowledge, risk perception, and perceived information-gathering capacity (PIGC) are all significant predictors of information needs [30].

Communication and interaction are critical components of emergency management. Access to information is critical for minimizing damage and casualties in disasters. Using global warming as an example, Kahlor et al. assessed attitudes toward behaviors, perceived behavioral control, and behavioral intent as new additions to the RISP model [26]. That study found that perceived social pressures have a significant impact when individuals seek disaster-related information. It also indicated that risk communicators should focus on how little the audience currently knows, how much they need to know, and how informed others expect them to be. This makes risk communication activities more effort intensive. Kellens et al. conducted an empirical study about how disaster victims search for risk information in flood disasters using three variables: risk perception, information needs, and self-efficacy. The classification of residents found that risk perception level and perceived hazard knowledge were higher for permanent residents compared to temporary residents, leading to increased information seeking behavior among permanent residents [29].

Several studies have found differences in information seeking behavior and channels between different types of disasters. For instance, tornado survivors report being alerted by others [31,32], television [33,34], and motor sirens [34,35]. For storms, key information sources include radio, friends and neighbors, and television. Broadcast media is the most important information channel before and during disasters when essential services and electricity are interrupted [36]. Peers serve as main and confirmatory sources during bushfires; environmental cues, radio, and agency websites are also important [37]. For terrorist attacks [38], tsunami, and earthquakes [39,40,41], television and radio are the main sources. Overall, in disasters, public sources of disaster information, such as television and Internet news websites, are more trusted than private sources, such as one’s family, friends, and social networking connections. With the recent development of information and communication technologies (ICTs), social media has also become an important information channel when people face disasters.

Many studies have investigated information seeking behavior based on different disaster types. Research remains fragmented, however, with respect to the specific phases of disasters, from prevention, to preparedness, response, and recovery. Some studies have focused on the disaster preparedness phase [42] and response phase [43,44]. However, disaster recovery and resettlement are critical phases for enhancing the region’s adaptation and resilience to disaster, and there has been little research on information seeking behavior during these phases. This is the focus of this paper.

Based on the studies above, there are several determinants of information seeking behavior worth investigating. Crucial determinants include individual characteristics (e.g., age, gender, hazard experience), perceived hazard characteristics, information insufficiency, channel beliefs, and information gathering capacity. Researchers also commonly add self-efficacy to the original RISP model. 

### 2.2. Determinants of Information Seeking Behavior

• Relevant Channel Belief

Channel beliefs refer to the degree to which an individual believes in an information channel. It relates to enthusiasm for using the channel, and considers both credibility and usefulness. The more people trust a channel, the more often they will use it when needed. Huurne and Gutteling found that channel beliefs were positively correlated with the willingness to search for information [9]. The stronger the public’s trust in information channels was, the more positive the public’s information-searching behavior was. Moreover, the more the public believes in a certain channel, the more they trust its ability to provide information accurately and effectively. As such, this study explored whether relevant channel beliefs positively affected information seeking behavior.

• Information Sufficiency

Information sufficiency, also known as information needs, plays a central role in risk communication [27]. This variable is widely regarded as a key motivator to seek risk information. Based on the sufficiency principle in the HSM, Griffin et al. defined information insufficiency as the perceived “gap” between current knowledge and sufficient knowledge (i.e., the threshold that one perceives as being sufficient) [10]. A lack of risk information drives people to devote more effort to find risk and disaster information. Information sufficiency is generally considered a negative predictor for information seeking behavior; if I believe I have enough information, I am less likely to seek additional information.

• Perceived Hazard Characteristics

Perceived hazard characteristics refer to the perception level of the risks themselves. This variable emphasizes the individual’s perception level of objective phenomena, and the individual’s direct perception and subjective risk judgment [45]. The study of public risk perception and perceived hazard characteristics has grown significantly over recent decades, and is now represented in nearly every risk domain. Paul Slovic originally demonstrated a set of higher-order characteristics that reflected the degree to which a risk is understood and the degree to which it evokes a “feeling of dread” [46]. Griffin’s model adjusts variables to represent perceptions of personal risk and the seriousness of risk outcomes [11]. Some key elements have been applied with respect to predicting information seeking behavior. Huurne referred to perceived hazard characteristics and affective responses as indirect predictors, indirectly influencing people’s motivation to seek risk information as a result of information insufficiency [27]. Choo proposed that perceived hazard characteristics would affect people’s judgment on the adequacy of their own information, leading to relevant information search behaviors [47]. Kellens et al. used risk information seeking behavior related to a coastal flood as a case study, and discussed the intermediary role that information insufficiency plays between perceived hazard characteristics and information seeking behavior [29]. That study also highlighted that the higher the level of risk perception is, the more information the public needs. This results in more frequent information seeking behavior. The less people know about a disaster, or the higher they perceive the required knowledge level to be, the higher their need for risk-related information will be. This leads to a higher intention to seek additional information.

• Self-Efficacy

Risk communication researchers have stressed the importance of considering one’s self-efficacy, or the belief that one can understand and execute certain actions to protect oneself from harm in the face of disasters. Kievik and Gutteling defined self-efficacy as the ability to successfully address a threat by seeking information that will help the person take adequate self-protective measures. They also proposed that self-efficacy is a trigger that can stimulate the public’s motivation to seek risk information [48]. Kellens et al. reported that self-efficacy was negatively correlated with information seeking behavior [29]. Bronstein further noted that self-efficacy is a key factor influencing information seeking behavior, which runs through the entire information seeking process [49]. The public usually lacks necessary knowledge about disasters and risks; as such, they have a low sense of self-efficacy. This can lead to helplessness and anxiety and the tendency to show more information seeking behavior.

• Information-Gathering Capacity

When an individual starts to learn more about a risk, information-gathering capacities are required [10,11]. A person with information-gathering capacity has three characteristics: an awareness that information can solve problems; a deep understanding of the necessity of information sources; and a good search strategy and method for collecting information [50]. Previous studies have found that perceived information-gathering capacity was an influential factor driving information seeking behavior. The stronger the perceived information-gathering capacity was, the more confident people were that they could get the needed information, and the stronger their desire was to search for the information [20,29,48]. This study also explored whether the information-gathering capacity positively affected information needs.

### 2.3. Hypotheses Development

**Figure 2:**
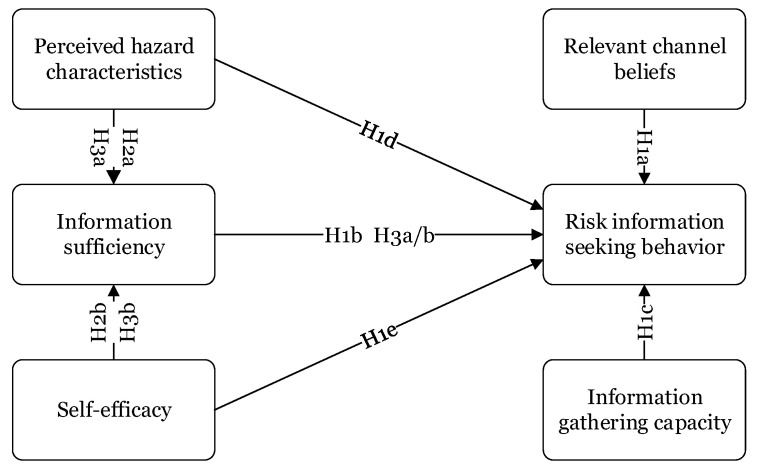
Hypotheses of risk information seeking behavior.

Based on the determinants and their relationships discussed above, Figure 2 describes a theoretical model of information seeking behavior. The following hypotheses were used to structure the model analysis. All of these hypotheses were tested in a disaster resettlement context.

**Hypothesis** **1a** **(H1a).** *Relevant channel beliefs positively relate to risk information seeking behavior;*

**Hypothesis** **1b** **(H1b).** *Information sufficiency negatively relates to risk information seeking behavior;*

**Hypothesis** **1c** **(H1c).** *Information-gathering capacity positively relates to risk information seeking behavior;*

**Hypothesis** **1d** **(H1d).** *Perceived hazard characteristics positively relate to risk information seeking behavior;*

**Hypothesis** **1e** **(H1e).** *Self-efficacy negatively relates to risk information seeking behavior;*

**Hypothesis** **2a** **(H2a).** *Perceived hazard characteristics negatively relate to information sufficiency;*

**Hypothesis** **2b** **(H2b).** *Self-efficacy positively relates to information sufficiency;*

**Hypothesis** **3a** **(H3a).** *Information sufficiency mediates the relationship between perceived hazard characteristics and risk information seeking behavior (H1d);*

**Hypothesis** **3b** **(H3b).** *Information sufficiency mediates the relationship between self-efficacy and risk information seeking behavior (H1e).*

## 3. Methodology

### 3.1. Study Area

**Figure 3:**
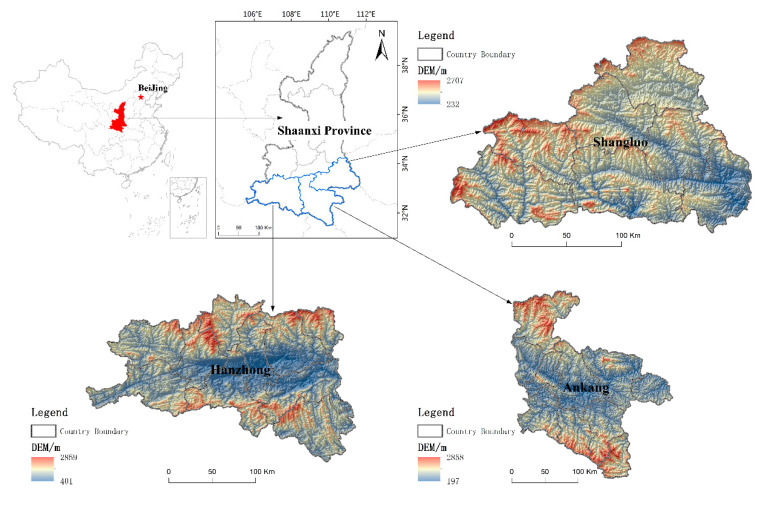
Area involved in the Resettlement of South Shaanxi (RSS).

China has the largest population in the world, a fragile ecological environment, and frequent natural hazards (e.g., earthquakes, floods, and mudslides). As such, it faces the arduous task of disaster mitigation. In addition to conventional measures, such as reservoir and dam construction, the Chinese government launched a disaster mitigation and preparedness program in 2011 called the Resettlement of South Shaanxi (RSS), which has been the largest disaster resettlement in the history of the country. The state requires governments at all levels to ensure that relocated people are actually willing to move. In addition, the state provides support policies and commercial loans, and encourages people to make flexible choices with respect to the time and method of relocation. Some studies [51,52,53] have described a continuum of "induced voluntarism" and "coercive voluntarism". According to the original RSS plans, 380,000 local households and approximately 1,400,000 people were to be resettled by the end of 2015. In addition, around 600,000 local households (2,440,000 residents) will be resettled by the end of 2020. Overall, people from 6253 villages, 26.38 per cent of the population in the south of Shaanxi Province, are involved in the program [54]. This program helps the cities of Ankang, Hanzhong, and Shangluo, located in the hinterland of the Qinling Mountains, south of Shaanxi Province, China, which have experienced serious flood- and earthquake-related disasters (see Figure 3). The RSS is different from the relocation caused by project construction. Its goal is to improve the sustainable development level of the region. In addition, local authorities believe that the RSS restores key ecosystem services in these areas, alleviates poverty, improves the livelihoods of rural households, and promotes rapid economic growth and urbanization. More detailed information on this specific program is available in a series of studies by Guo and Kapucu [2,3,4].

However, the RSS remains controversial. *The New York Times* in the United States reported some problems, such as increased unemployment, as a result of resettlement. After losing farmland, peasants usually need more resources to maintain their family livelihoods after resettlement [55]. It is not possible to meet all the demands of the population; as a result, conflicts may occur during and after resettlement. When faced with such problems, they seek help from neighbors, friends, intellectuals, legal professionals, house developers, and local entrepreneurs, or they seek information on social media. More recently, especially during resettlement community development, the participants in the conflict have also included village committees and village officials. A lack of effective communication or negotiation can lead to confrontations between peasants and local governments, resulting in injury and even death. As such, the RSS was selected as a case study example.

Data were collected using surveys from resettlement area respondents between June and August 2019. Respondents from four resettlement sites of Ziyang County, Ankang City, Shaanxi Province provided their practical feedback in the form of survey responses. Ziyang County has a high poverty level in China, and is one of the largest resettled areas in the RSS. Since the RSS was introduced in 2011, 37,200 households and 129,000 people in Ziyang county have moved from inhospitable and disaster-prone areas. By 2018, Ziyang county had completed the relocation of 26,817 households and 93,226 people, and the employment resettlement rate reached 80%. However, residents perceive different degrees of the probability of risk and its potential consequences in the post-disaster resettlement process (both disaster risks and livelihood risks). This has led to social conflicts in the county [53,56]. The local authorities have focused on the resettlement of Ziyang County to solve the livelihood-related problems in the RSS and improve the local resilience.

### 3.2. Questionnaire Description

The anonymous questionnaire consisted of two parts. The first part collected information about the respondents’ gender, age, education level, annual household income, etc. The second part asked questions about information seeking behavior and its influencing factors in the RSS. Table 1 lists the items depicted in Figure 2. Some items, such as information seeking behavior, channel beliefs, information sufficiency, information-gathering capacity, and perceived hazard characteristics, were measured based on previous empirical studies; however, the items related to self-efficacy were new. Table 1 provides the specific items in the survey. The structured survey questionnaire included 28 closed-ended questions, assessed using a five-point Likert scale, ranging from strongly disagree to strongly agree as 1–5, respectively.

### 3.3. Implementation

Questionnaires were collected from four resettlement sites in Ziyang County between June and August 2019: Songba (Site A), Jingjia (Site B), Lanjiaping (Site C), and Xiangyang (Site D). These four sites have many residents, most of whom are long-term residents with an average age distribution, achieving broad participant coverage. In addition, these sites have well-constructed public facilities and community management agencies.

First, to verify the validity of the questionnaire, we first conducted field research. A total of 200 questionnaires were distributed in the early stage for pre-investigation. The questionnaire was subsequently revised and a total of 800 questionnaires were distributed using the quota sample method. A total of 682 questionnaires were completed, resulting in a response rate of 85.25%. There were 258 questionnaires from Site A, 100 from Site B, 202 from Site C, and 122 from Site D. After screening the returned surveys, 616 valid questionnaires remained. There were two possible reasons for the high response rate. The first reason was the recent flood and geological disasters (such as the Wenchuan earthquake in 2008 and Jiuzhaigou earthquake in 2017), making the survey highly relevant to residents’ daily lives. The second reason was that during the survey, the collected questionnaires were reviewed in real time. If any blank items were found, the respondents were asked to fill in the blank items.

### 3.4. Analysis Steps

The statistical analysis was conducted using the following four steps: (1) a frequency analysis was used to show the characteristics of respondents. (2) An exploratory factor analysis (EFA) was used to extract the dimensions, and then a Kaiser–Mayer–Olkin (KMO) test was implemented to test whether the aforementioned EFA was appropriate. Then, the reliability and validity were evaluated by calculating Cronbach’s α coefficient. (3) Confirmatory factor analysis (CFA) was analyzed to assess the stability of the relationship between the structural factors and EFA measurement items, and to identify the presence of cross validity. (4) A structural equation model (SEM) was used to test the proposed conceptual model on disaster resettlement risk information seeking behavior. 

## 4. Results

### 4.1. Characteristics of Respondents

Of the survey respondents, there was a higher proportion of women than men, with women accounting for 60.1% of the sample. In the resettlement area, most men go to work, so there were slightly more women staying in the resettlement sites to respond to the survey. Among the survey respondents, participants aged 31 and above accounted for 77.6% of the total. This is because most young people go to work, leaving mainly middle-aged and elderly people as the available respondents. Approximately 86.6% of the participants had a junior high school education or below, and 57.1% of respondents earned less than RMB 8000 (approximately USD 1128) per family per year.

### 4.2. Exploratory Factor Analysis and Reliability Analysis

After EFA was used to extract the dimensions, the results of the KMO test and Bartlett’s test of sphericity indicated that KMO almost exceeded 0.70 (see Table 2). This indicated that the set of data was suitable for the EFA [39]. All 28 items had a factor loading greater than 0.5, which corresponded to the six dimensions (see Table 1). As Table 2 shows, the Cronbach’s α values ranged from 0.677 to 0.790, higher than the threshold value of 0.60. Internal consistencies were also satisfactory for the six constructs.

### 4.3. Confirmatory Factor Analysis

Table 3 shows that construct loadings were highly statistically significant (*p* < 0.001, exceeding the threshold value of 0.50) [61]. This indicated that the latent variables were explained by the measured variables.

### 4.4. Structural Model and Hypothesis Testing

**Figure 4:**
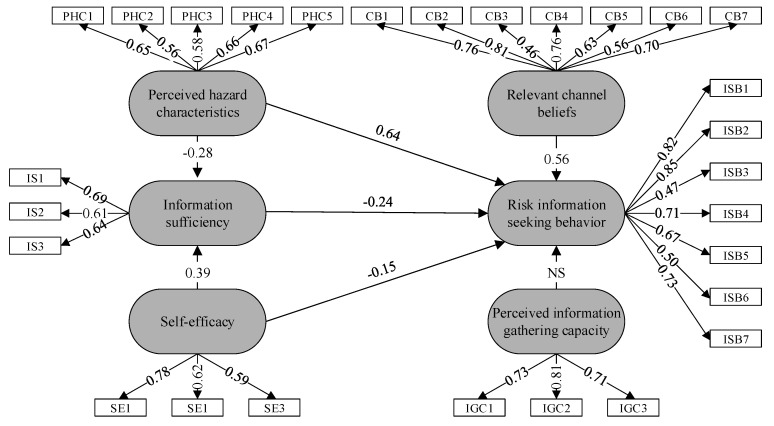
Path coefficients of the structural equation model (SEM).

After the CFA, the structural model was assessed using model fit measurements. The model fit was at an acceptable level (see Table 4). Table 5 and Figure 4 show the results of the causal relationships and correlations of each path, respectively.

#### 4.4.1. Descriptive Statistics of Risk Information Seeking Behavior in the RSS

For the first research goal, Table 6 shows that, based on the mean value and square deviation of the seven information channels observed, respondents reported investing different levels of effort to obtain relevant risk information through different channels. The mean values of the seven information channels, from the highest to the lowest, for village committees and officials, friends and neighbors, relatives and family, government officials, news broadcasts, resettlement house developers and local entrepreneurs, and social media (Weibo, WeChat, Internet forums, etc.), were 3.69, 3.46, 3.43, 3.31, 3.18, 2.83, and 2.57, respectively. Respondents reported mainly obtaining information related to disaster resettlement from village committees and officials, followed by neighbors, friends, and family members and relatives. The proportion of information obtained from village committees and officials was significantly higher compared to the other six channels. Fewer respondents reported seeking resettlement information through information and communication technologies (ICTs) such as Weibo and WeChat and other social media tools. Overall, the respondents’ information seeking behavior was relatively positive, reflecting the public’s high attention to disaster resettlement measures and regional resilience related to their own vital interests.

Respondents reported significant differences in their beliefs about the different information channels. The mean values of the seven channels, from the highest to the lowest, for news broadcasts, relatives and family, village committees and officials, friends and neighbors, government officials, resettlement house developers and local entrepreneurs, and social model tools, were 3.84, 3.69, 3.67, 3.64, 3.52, 3.23, and 2.59, respectively. As such, news broadcasting was the channel most trusted by respondents.

In terms of information sufficiency, the highest one related to the pros and cons of resettlement sites. The scope of resettlement ranked second. However, based on the absolute value of the mean value, respondents reported that information about the three categories of IS1 (relates to the scope of resettlement and relocation information), IS2 (relates to the pros and cons of each resettlement site), and IS3 (relates to the supporting policies and measures related to disaster resettlement) is all insufficient. This means that respondents perceive not knowing enough about the information related to the RSS, and perceive the possible harm, risk, and future impacts of RSS as unclear.

In the information-gathering capacity, the order from high to low, is the capacity to obtain information (IGC1), the capacity to understand information (IGC2), and the capacity to evaluate information (IGC3). Since the mean values of all items are greater than 3, respondents had a high degree of recognition for their information-gathering capacities.

Perceived hazard characteristics emphasize an individual’s perception level of risks. As Table 6 shows, respondents had a higher level of risk perception, as the mean values of all items are greater than 3. As mentioned in Section 2.2, the higher the level of risk perception, the more disturbed the public will be, and the more information they will need. Among all the items, the respondents had the highest level of perception of risk persistence (PHC1), followed by the scientific knowledge of risks (PHC5), the reduction of risks (PHC2), the control of risks (PHC3), and the individual knowledge of risks (PHC4).

For self-efficacy, when the relevant policies and measures of RSS specifically affected respondents (often in a negative way that causes problems), most respondents reported not having the professional knowledge, financial ability, or time to solve the problems they may face. This is reflected in the relatively low mean values of SE1, SE2, and SE3, at 2.17, 2.36, and 2.54, respectively.

#### 4.4.2. Direct Effects in the Information Seeking Behavior Process of the RSS

For the second research goal, hypotheses H1a, H1b, H1c, H1d, and H1e were tested using the SEM. Figure 4 and Table 3 show that relevant channel beliefs significantly positively impact information seeking behavior (H1a), with a standardized coefficient of 0.56, *p* < 0.05. Consistent with previous studies [27], the higher the public’s trust in channels was, the more active the information seeking behavior was in the RSS. Information sufficiency significantly negatively influenced public information seeking behaviors in the RSS (H1b), with a standardized coefficient of −0.24, *p* < 0.05. The more the public grasped the information, the less active they were in seeking information in the RSS process. Perceived hazard characteristics had a significantly positive effect on public information seeking behavior (H1d), with a standardized coefficient of 0.64, *p* < 0.001. The higher the public’s perception of the possible risks brought by RSS was, the more likely they were to conduct information seeking behavior to avoid risks. Self-efficacy had a significantly positive effect on information seeking behavior in the RSS, which is consistent with the results of Bronstein et al. (H1e), with a standardized coefficient of −0.15, *p* < 0.001. The higher the self-efficacy of the public was, the less active the information seeking behavior was.

However, information-gathering capacity had non-significant direct influences on public information seeking behavior in the RSS (H1c), with a standardized coefficient of −0.05. These direct relationships have not been consistently found in previous research. 

#### 4.4.3. Mediating Effect in the Information Seeking Behavior Process of the RSS

This study also explored the mediating effect of information sufficiency. First, perceived hazard characteristics had a significantly negative effect on information sufficiency (H2a), with a standardized coefficient of −0.28, *p* < 0.001; information sufficiency significantly negatively influenced public information seeking behavior (H1b), with a standardized coefficient of −0.24, *p* < 0.05; and perceived hazard characteristics had a significantly positive effect on public information seeking behavior (H1d), with a standardized coefficient of 0.64, *p* < 0.001. Therefore, information sufficiency had a mediating role between perceived hazard characteristics and information seeking behaviors, supporting H3a. Additionally, self-efficacy significantly positively affected information sufficiency (H2b), with a standardized coefficient of 0.39, *p* < 0.001; information sufficiency significantly negatively influenced public information seeking behavior (H1b), with a standardized coefficient of −0.24, *p* < 0.05; and self-efficacy significantly positively affected information seeking behavior (H1e). As such, information sufficiency was found to play a mediating role between self-efficacy and information eeking behaviors, supporting H3b.

## 5. Conclusions

This study focused on the public’s information seeking behavior in the context of resettlement in South Shaanxi, China. Based on previous information seeking models, the study tested empirical relationships between information seeking behavior and other determinants. The mediating role of information sufficiency has also been examined in the information seeking process. A total of 616 valid surveys were collected from respondents in Ziyang County in Ankang City, China. A structural equation model was used to statistically test several hypotheses, reflecting the different relationships of the hypothesized model in Figure 2. This section discusses the conclusions and implications.

Firstly, there is currently one primary channel to support resident information seeking in the RSS, which mainly relies on the village committees and officials. Moreover, residents invest significantly greater effort compared to other methods to seek information through this channel. ICTs, such as Weibo and WeChat, are underutilized. The main channel for information seeking, however, differs from the level of trust associated with the channel. In addition, there is a mismatch between the primary channel and the most trusted channel associated with information seeking behavior. The main channel for information is village committees and officials; however, residents trust news broadcasts most. This deviation often significantly affects the efficiency of information dissemination and the reliability of information content, hindering the effective circulation of RSS information. 

Secondly, relevant channel beliefs, information sufficiency, perceived hazard characteristics, and self-efficacy significantly directly influence risk information seeking behavior in the RSS: (1) relevant channel beliefs significantly positively impact information seeking behavior. According to the specific situation of the RSS, the public has a higher level of trust in news broadcasts, relatives and family, and village committees and officials. The study found the public is more willing to obtain RSS-related information using the above traditional information channels and is willing to invest time, money, and effort to obtain that information. In the process of visiting residents, we found that this result was mainly due to the low acceptance and utilization rate of modern information channels and the insufficiency of network infrastructure in rural areas. (2) Information sufficiency significantly negatively influences public information seeking behaviors in the RSS. This result aligns with previous research. The more thoroughly the public understands the content and possible problems with RSS-related policies and measures, and the more fully the relevant background information is obtained, the fewer efforts they will make to seek information. (3) Perceived hazard characteristics significantly positively impact public information seeking behavior. In implementing RSS-related policies and measures, the public will actively search for possible risks or harms caused by the RSS to avoid losses caused by a lack of information. Moreover, the stronger the sense of crisis and insecurity is, the more active and positive information seeking behavior is. This is embodied in the increasing frequency of searching for and exploring policy supporting measures, compensation standards, and future planning of the RSS. (4) Self-efficacy has a significantly positive effect on information seeking behavior in the RSS. The stronger the public’s sense of control over the possible risks brought by the RSS is, and the more confident they are in dealing with the risks, the lower the frequency of information seeking behavior, and the less effort they will invest in information seeking behavior.

However, information-gathering capacity has non-significant direct influences on information seeking behavior of the RSS which have not been consistently found in previous research. There are two possible reasons for this: (1) previous studies mainly focused on potential risk settings. Ziyang County in the RSS was selected as the focus of the study because it is located in a plate fault zone, and has experienced numerous disasters, including earthquakes, floods, and mudslides. Local governments and relevant departments have made continuous efforts to communicate disaster information and emergency preparedness plans to local residents through different channels. The rich information environment has led some residents to feel they have enough knowledge about disaster avoidance and resettlement. This leads to a reduced need for information and a cessation of information seeking behavior. This may explain why information-gathering capacity has a non-significant direct influence on the public information seeking behavior of the RSS. (2) With the increase in the frequency of earthquakes, floods, and mudslides and the change of resettlement policies, residents’ attitudes towards RSS have evolved from initial nervousness, worry, and anxiety to numbness and less attention. This has led to a decrease in information seeking activities.

Lastly, in the exploration of a mediating effect, perceived hazard characteristics and self-efficacy drive risk information seeking behavior in both direct and indirect ways through information sufficiency. In the RSS, respondents reported that the higher the level of perceived unknown risks and uncertainties is, the lower the level of information they have about RSS policies and future livelihood restoration measures. At the same time, under the threat of unknown risks and uncertainties, people are motivated to actively search for information, such as resettlement planning and subsidy standards. Additionally, when the respondents’ perceptions of the unknown risk of resettlement is certain, and if the respondents believe they can control the negative impact of the unknown risk to themselves and reasonably resolve it, then their confidence level is relatively high. This reduces their efforts to conduct information seeking behavior. Therefore, in contrast to other contexts [29,62], in the RSS, we conclude that self-efficacy and perceived hazard characteristics have an indirect effect on information seeking behavior in the RSS through information sufficiency.

Several implications emerge from this study: (1) post-disaster resettlement is an important step in improving the resilience of communities and regions. Resettlement protects residents from future disasters, including earthquakes and floods, and engages the sustainable development of residents’ livelihoods. Research results indicated that information sufficiency played a noteworthy mediating role in the RSS process. Therefore, disaster resettlement should pay more attention to information disclosure and risk communication during the process. (2) The difference between the information channels used by residents, and the channels most trusted by residents, was highlighted. This difference may greatly affect the efficiency of information dissemination and the reliability of information content in disaster resettlement. 

In the future, risk managers can make timely adjustments to information disclosure channels, based on the characteristics of the different stages of disaster management and risk governance. For example, in the emergency preparedness stage, information channels with large coverage and low costs can be used, such as village committee notices and posters and emergency training and lectures. During the emergency response stage, channels with strong persistence and high resident trust can be adopted. This includes news broadcasts, household notifications, and visits. In the disaster resettlement and recovery stage, sustainable and diversified information dissemination channels should be adopted, such as enhancing the use of ICT platforms. This would include paying attention to classification guidance, based on different residents’ information-gathering capacities, willingness, and preferences in choosing the most suitable channels.

Additionally, we recommend that communication strategies and content should also be focused on during the disaster resettlement process. In terms of communication strategies, as most resettlement occurs in rural areas, residents have a low level of knowledge and poor livelihoods. This greatly affects the ability to understand policy content, compensation schemes, and future planning. This, in turn, causes cognitive gaps and potential risks between the government and the public. Therefore, in the disaster resettlement process, especially in rural areas, risk managers should avoid using obscure official jargon and encourage mass media to promote more user-friendly images, videos, and other information via ICT platforms based on playing a leading role in traditional information channels, like news broadcasts. In terms of communication content, based on the results in Section 4.4.1, we recommend that it is beneficial to first publicize the advantages and disadvantages of the disaster resettlement, and then engage in specific and technical outreach, such as scope and geographical conditions. 

Like all studies, this research had some limitations, which should be considered when reviewing its conclusions and implications: (1) resettlements in Shaanxi Province involve Ankang City, Hanzhong City, and Shangluo City in the south of Shaanxi Province, and some regions in the north of Shaanxi Province. This study included only respondents in Ankang City; no other regions were involved. Future studies should consider other areas. (2) The study involved respondents in Ankang City in August 2019, but the estimated duration of the RSS is 10 years. Therefore, information seeking behavior by immigrant respondents has not been assessed during different stages of RSS. This is an opportunity for future studies. Despite these limitations, this study is important because it enriches the risk information seeking behavior and risk communication research in the context of disaster resettlement.

## Figures and Tables

**Table 1:** Constructs, items, and references.

Constructs	Items	References
**Information seeking behavior (ISB)**	To what extent do you intend to seek disaster resettlement information from your relatives and family (ISB1).	Newman and Staelin, 1971; Griffin, 1999; Li et al., 2017 [11,39,57]
	To what extent do you intend to seek disaster resettlement information from your friends and neighbors (ISB2).	
	To what extent do you intend to seek disaster resettlement information through village committees and village officials (ISB3).	
	To what extent do you intend to seek information through resettlement house developers and local entrepreneurs (ISB4).	
	To what extent do you intend to seek disaster resettlement information through the news broadcast (ISB5).	
	To what extent do you intend to seek disaster resettlement information from government officials (ISB6).	
	To what extent do you intend to seek disaster resettlement information through Weibo, WeChat ^1^, Internet forums, etc. (ISB7).	
**Relevant channel beliefs (CB)**	I trust the information channel of relatives and family (CB1).	Huurne and Gutteling, 2008; Dunwoody and Griffin, 2014 [9,58]
	I trust the information channel of friends and neighbors (CB2).	
	I trust the information channel of the village committee and the village officials (CB3).	
	I trust the information channel of resettlement house developers and local entrepreneurs (CB4).	
	I trust the information channel of the news broadcast (CB5).	
	I trust the information channel of government officials (CB6).	
	I trust the information channel of Weibo, WeChat, Internet forums, etc. (CB7).	
**Information sufficiency (IS)**	I understand the scope of resettlement and relocation information (IS1).	Huurne, 2008; Huurne and Gutteling, 2009 [27,59]
	I understand the pros and cons of each resettlement site (IS2).	
	I understand the supporting policies and measures related to disaster resettlement (IS3).	
**Information-gathering capacity (IGC)**	I can get information about resettlement policies as soon as I ask (IGC1).	Griffin et al., 2008; Kellens et al., 2012; Yang et al., 2014 [10,20,29]
	I think the disaster resettlement policy documents and plans are quite understandable (IGC2).	
	I have my own views on resettlement policy and related information (IGC3).	
**Perceived hazard characteristics (PHC)**	The government can solve the problem of resettlement quickly, and we have nothing to worry about (PHC1).	Peters and Slovic, 1996; O’Neill et al., 2016; Choo, 2017 [45,47,60]
	Problems in the process of resettlement will be resolved quickly without lasting impact (PHC2).	
	Disaster resettlement has produced some problems, but most of the problems will be solved (PHC3).	
	I understand the disaster resettlement may bring some risks (PHC4).	
	Experts have certain types of help for disaster resettlement policy formulation and the implementation process (PHC5).	
**Self-efficacy (SE)**	If disaster resettlement impacts me, I am willing to understand or solve them, even if it means spending some time (SE1).	Kievik and Gutteling, 2011; Kellens et al., 2012; Bronstein, 2014 [29,48,49]
	If disaster resettlement impacts me, I am willing to understand or solve them, and even spend some money (SE2).	
	For disaster resettlement, I have some professional knowledge about disaster decision-making, planning and construction, etc. (SE3).	

^1^ Weibo is one of the biggest social media platforms in China. WeChat is a mobile text and voice messaging communication service developed by Tencent in China.

**Table 2:** Summary of exploratory factor analysis and reliability analysis.

Value	Information Seeking Behavior	Relevant Channel Beliefs	Information Sufficiency	Information-Gathering Capacity	Perceived Hazard Characteristics	Self-Efficacy
Cronbach’s α	0.777	0.783	0.679	0.790	0.788	0.677
KMO	0.764	0.830	0.671	0.702	0.802	0.667

KMO: Kaiser–Mayer–Olkin test.

**Table 3:** Overall confirmatory factor analysis for the measurement model.

Items	Measurement Items	Construct Loadings
Information seeking behavior	ISB1	0.80 (***)
	ISB2	0.86 (***)
	ISB3	0.45 (***)
	ISB4	0.70 (***)
	ISB5	0.62 (***)
	ISB6	0.53 (***)
	ISB7	0.69 (***)
Relevant channel beliefs	CB1	0.74 (***)
	CB2	0.82 (***)
	CB3	0.56 (***)
	CB4	0.75(***)
	CB5	0.53 (***)
	CB6	0.71 (***)
	CB7	0.48 (***)
Information sufficiency	IS1	0.65 (***)
	IS2	0.63 (***)
	IS3	0.65 (***)
Information-gathering capacity	IGC1	0.72 (***)
	IGC2	0.80 (***)
	IGC3	0.69 (***)
Perceived hazard characteristics	PHC1	0.69 (***)
	PHC2	0.66 (***)
	PHC3	0.68 (***)
	PHC4	0.63 (***)
	PHC5	0.60 (***)
Self-efficacy	SE1	0.79 (***)
	SE2	0.59 (***)
	SE3	0.54 (***)
df = 2.608, RMSEA = 0.07, NFI = 0.833, CFI = 0.830, GFI = 0.843, AGFI = 0.808.		

df: degrees of freedom; RMSEA: root mean square error of approximation; NFI: normed fit index; CFI: comparative fit index; GFI: goodness of fit index; AGFI: adjusted goodness of fit index. Notes: *** *p* < 0.001. We believe the values of RMSEA and CFI meet the standards in this study. Although some research suggested strict thresholds such as a CFI value over 0.90 and an RMSEA value less than or equal to 0.06, in reality, this is difficult to achieve.

**Table 4:** The summary of model fit indices.

Fit Index	χ^2^/df	RMSEA	*p*	CFI	NNFI	GFI	AGFI
Value	2.836	0.077	0.000 (***)	0.806	0.779	0.824	0.785

**NNFI**: non-normed fit index. Notes: *** *p* < 0.001. We believe the values of RMSEA and CFI meet the standards in this study. Although some research suggested strict thresholds such as a CFI value over 0.90 and an RMSEA value less than or equal to 0.06, in reality, this is difficult to achieve.

**Table 5:** Results of the hypothesis testing.

Path	Proposed Direction	Standardized Coefficient	Result
H1a: Relevant channel beliefs→risk information seeking behavior	+	0.56 (**)	Accepted
H1b: Information sufficiency→risk information seeking behavior	-	−0.24 (**)	Accepted
H1c: Information-gathering capacity→risk information seeking behavior	+	−0.05	Rejected
H1d: Perceived hazard characteristics→risk information seeking behavior	+	0.64 (***)	Accepted
H1e: Self-efficacy→risk information seeking behavior	-	−0.15 (***)	Accepted
H2a: Perceived hazard characteristics→ information sufficiency	-	−0.28 (***)	Accepted
H2b: Self-efficacy→information sufficiency	+	0.39 (***)	Accepted
H3a: Perceived hazard characteristics→information sufficiency→risk information seeking behavior			Accepted
H3b: Self-efficacy→information sufficiency→risk information seeking behavior			Accepted

Notes: ** *p* < 0.05, *** *p* < 0.001.

**Table 6:** Descriptive statistical results of data.

Items	Mean (Order)	Standard Deviation
**1. Information seeking behavior**	22.47	8.93
ISB1	3.43 (3)	1.01
ISB2	3.46 (2)	1.05
ISB3	3.69 (1)	1.21
ISB4	2.83 (6)	1.46
ISB5	3.18 (5)	1.15
ISB6	3.31 (4)	1.40
ISB7	2.57 (7)	1.65
**2. Relevant channel beliefs**	24.18	8.67
CB1	3.67 (3)	0.94
CB2	3.64 (4)	0.97
CB3	3.69 (2)	0.94
CB4	3.23 (6)	1.34
CB5	3.84 (1)	1.47
CB6	3.52 (5)	1.07
CB7	2.59 (7)	1.93
**3. Information sufficiency**	7.42	3.02
IS1	2.53 (2)	1.03
IS2	2.54 (1)	1.08
IS3	2.35 (3)	0.91
**4. Information-gathering capacity**	9.25	4.56
IGC1	3.18 (1)	1.46
IGC2	3.06 (2)	1.54
IGC3	3.01 (3)	1.56
**5. Perceived hazard characteristics**	18	4.22
PHC1	3.67 (1)	0.87
PHC2	3.60 (3)	0.86
PHC3	3.58 (4)	0.87
PHC4	3.54 (5)	0.85
PHC5	3.61 (2)	0.78
**6. Self-efficacy**	7.07	2.31
SE1	2.17 (3)	0.64
SE2	2.36 (2)	0.73
SE3	2.54 (1)	0.94
